# Neuronal Ceroid Lipofuscinosis Type 6 (CLN6) clinical findings and molecular diagnosis: Costa Rica’s experience

**DOI:** 10.1186/s13023-021-02162-z

**Published:** 2022-01-10

**Authors:** R. Badilla-Porras, A. Echeverri-McCandless, J. M. Weimer, A. Ulate-Campos, A. Soto-Rodríguez, A. Gutiérrez-Mata, L. Hernández-Con, S. Bogantes-Ledezma, A. Balmaceda-Meza, J. Brudvig, A. Sanabria-Castro

**Affiliations:** 1 Clinical Genetic and Metabolism Department, National Children’s Hospital, CCSS, San José, Costa Rica; 2 Research Unit, Hospital San Juan de Dios, CCSS, San José, Costa Rica; 3 Pediatrics and Rare Diseases Group, Sanford Research, Sioux Falls, SD USA; 4 Neurology Department, National Children’s Hospital, CCSS, San José, Costa Rica; 5 CENDEISSS, CCSS, San José, Costa Rica; 6 Pharmacology Department, Pharmacy School, Universidad de Costa Rica, San José, Costa Rica

**Keywords:** Neuronal ceroid lipofuscinosis, Batten disease, CNL6, Variant late infantile NCL, vLINCL, Lysosomal storage disease

## Abstract

**Background:**

Commonly known as Batten disease, the neuronal ceroid lipofuscinoses (NCLs) are a genetically heterogeneous group of rare pediatric lysosomal storage disorders characterized by the intracellular accumulation of autofluorescent material (known as lipofuscin), progressive neurodegeneration, and neurological symptoms. In 2002, a disease-causing NCL mutation in the CLN6 gene was identified (c.214G > T) in the Costa Rican population, but the frequency of this mutation among local Batten disease patients remains incompletely characterized, as do clinical and demographic attributes for this rare patient population.

**Objective:**

To describe the main sociodemographic and clinical characteristics of patients with a clinical diagnosis for Batten Disease treated at the National Children's Hospital in Costa Rica and to characterize via molecular testing their causative mutations.

**Methods:**

DNA extracted from buccal swabs was used for CLN6 gene sequencing. Participants’ sociodemographic and clinical characteristics were also obtained from their medical records.

**Results:**

Nine patients with a clinical diagnosis of Batten disease were identified. Genetic sequencing determined the presence of the previously described Costa Rican homozygous mutation in 8 of 9 cases. One patient did not have mutations in the CLN6 gene. In all cases where the Costa Rican CLN6 mutation was present, it was accompanied by a substitution in intron 2. Patients were born in 4 of the 7 Costa Rican provinces, with an average onset of symptoms close to 4 years of age. No parental consanguinity was present in pedigrees. Initial clinical manifestations varied between patients but generally included: gait disturbances, language problems, visual impairment, seizures and psychomotor regression. Cortical and cerebellar atrophy was a constant finding when neuroimaging was performed. Seizure medication was a common element of treatment regimens.

**Conclusions:**

This investigation supports that the previously characterized c.214G > T mutation is the most common causative NCL mutation in the Costa Rican population. This mutation is geographically widespread among Costa Rican NCL patients and yields a clinical presentation similar to that observed for CLN6 NCL patients in other geographies.

## Background

Neuronal ceroid lipofuscinoses (NCLs) constitute a group of genetic neurodegenerative diseases associated with motor and cognitive regression; progressive cortical, thalamic, and cerebellar atrophy; retinopathy; epilepsy; and a shortened lifespan [1, 2]. NCLs causative mutations have been mapped to at least 13 different genes, which encode a diverse set of lysosomal enzymes, cytosolic chaperones, and transmembrane proteins with roles in secretory and endolysosomal trafficking [3]. More than 70 mutations have been reported in the *CLN6* gene, which are responsible for variant late infantile or adult-onset forms (i.e. Kufs Disease Type A) of NCL [4–6]. The *CLN6* gene is located on 15q23 and encodes a 311 amino acid transmembrane endoplasmic reticulum protein that functions in the ER to Golgi transfer of lysosomal enzymes [7]. Downstream cellular pathologies ranging from synaptic alterations, glial activation, and autophagy have also hinted at additional cellular roles or, perhaps, specialized roles in neurons and glia [2, 6, 8–12].

The initial clinical manifestations of CLN6 disease generally begin between the ages of 2 and 4 years [13] and are characterized by motor regression, visual loss, the presence of rapid and involuntary muscle movements, and multiple seizures types [14, 15]. In advanced stages, patients commonly develop ataxia, further cognitive and motor deterioration, and finally spastic quadriparesia [16, 17]. Imaging-based studies commonly find cortical and/or subcortical grey matter atrophy [15, 17–19] and, like most other forms of NCL, there are no approved disease-modifying treatments. Clinical treatment is focused on symptom management and includes the management of seizures, sleep alterations, extrapyramidal symptoms, behavioral disturbances, anxiety and psychosis [14]. Slight variations in the clinical course of CLN6 disease have been linked to the causative mutations and the country of origin [20].

Mutations in the *CLN6* gene are observed mainly in patients of Portuguese, Indian, Pakistani or Czech descent, but have also been documented in countries such as Costa Rica, Sudan, Turkey and Japan [16, 21–23]. By the end of the 1990s, studies aimed at identifying and analyzing the most common genetic variant in late infantile NCL began in the Costa Rican population [24–26]. From these studies, the causative alteration known as the Costa Rican *CLN6* gene mutation was defined [27]. This genetic modification, described in 2002, corresponds to a nonsense change in exon 3 (c.214G> T; p.E72X) introducing a stop codon at amino acid position 72; which results in a premature termination and generates an incomplete and non-functional protein product that is degraded at the level of the endoplasmic reticulum [6, 22]. Other possible disease-related mutations in the *CLN6* gene (specifically in exons 4 [c.368G> A] and 7 [c.722T> C]) have also been reported [28]. Currently in Costa Rica, a NCL diagnosis is made by a clinical evaluation of the symptoms and signs recalled during medical history and present during physical examination by a pediatric neurologist.

Here, we aimed to determine the frequency of the c.214G>T *CLN6* gene mutation and any other mutations among Costa Rican NCL patients and to describe the main sociodemographic and clinical characteristics in patients diagnosed with NCL in the National Children's Hospital (HNN), the only pediatric hospital in Costa Rica, and part of the social security system (CCSS).

## Methods

After obtaining approval from the Institution’s Scientific Ethics Committee (CEC-CENTRAL-CCSS) and authorization from the Director of Costa Rica´s National Children´s Hospital (HNN), the parents of all patients with a clinical diagnosis of Batten disease currently treated at the HNN Neurology Department were contacted, the study was explained and the investigators invited the parents so their child could participate. Subsequently, an appointment was scheduled in the HNN and, after explaining the study and obtaining informed consent (informed assent was not possible), the investigator collected a buccal swab for each participant. The samples were disassociated from any personal identification and transported in compliance with international regulations to the appointed laboratory. The genetic analysis of the samples was performed by Sanford Research (Sioux Falls, SD; USA) in two independent laboratories where DNA was extracted and next generation sequencing (NGS) for the *CLN6* gene was performed for each study subject. Once the genetic results were obtained, the study participant´s parents received the genetic testing results with an explanation as part of their usual clinical visit.

The main sociodemographic and clinical characteristics from the study subjects were examined with a descriptive analysis based on existing medical records. These included: age, sex, place of birth and residence, age of initial symptom onset, age of clinical diagnosis, relevant medical history, main clinical manifestations and pertinent evaluations previously performed (ophthalmological evaluation, visual evoked potentials, electroretinogram, electroencephalogram, imaging tests including computerized axial tomography or brain magnetic resonance imaging) at diagnosis, in addition to the result of the most recent neurological examination and current treatment.

All study procedures were carried out in accordance with the Declaration of Helsinki, Good Clinical Practice Standards and local Costa Rican regulations.

## Results

Nine patients with a previous clinical diagnosis of NCL were identified. The average age of the subjects when the DNA sample was obtained was 9.9 ± 2.7 years (mean ± SD). The initial symptoms appeared at 3.9 ± 0.6 years on average (range 3-5 years) and the typical age at the time of the clinical diagnosis was approximately 7 years (range 3-10 years). Females outnumbered males in a 2:1 ratio. Most patients (77.8%, n = 7) did not report a significant medical history before the onset of symptoms related to the disease.

The Costa Rican mutation (homozygous nonsense variant in exon 3 c.214G> T in the CNL6 gene) was reported in approximately 90% (n = 8) of study participants. In all cases where the Costa Rican CLN6 mutation was present, it was accompanied by a substitution in intron 2 (c.198 + 104T>C, Table 1) as previously described [4, 22].

**Table 1:** Patient´s main sociodemographic characteristics, medical history and genetic mutation

Patient codification	A	B	C	D	E	F	G	H	I
Age	11	13	13	7	12	6	8	8	11
Sex	Male	Female	Female	Male	Female	Female	Female	Female	Male
Age of onset	4	ND	5	4	3	4	4	4	3
Age of diagnosis	ND	ND	ND	ND	10	6	8	8	3
Relevant medical history	Severe myopia	Severe TBI w/neurological sequelae	None	None	None	None	None	None	None
Genetic	NI	Homozygous	Homozygous	Homozygous	Homozygous	Homozygous	Homozygous	Homozygous	Homozygous
Mutation	E3: NI	E3: c.214 G > T	E3: c.214 G > T	E3: c.214 G > T	E3: c.214 G > T	E3: c.214 G > T	E3: c.214 G > T	E3: c.214 G > T	E3: c.214 G > T
	I2: NI	I2: c.198 + 104 T > C	I2: c.198 + 104 T > C	I2: c.198 + 104 T > C	I2: c.198 + 104 T > C	I2: c.198 + 104 T > C	I2: c.198 + 104 T > C	I2: c.198 + 104 T > C	I2: c.198 + 104 T > C
	E3: NI	E3: p.E72X	E3: p.E72X	E3: p.E72X	E3: p.E72X	E3: p.E72X	E3: p.E72X	E3: p.E72X	E3: p.E72X

*ND* no data, *TBI* traumatic brain injury, *NI* not Identified

**Fig. 1:**
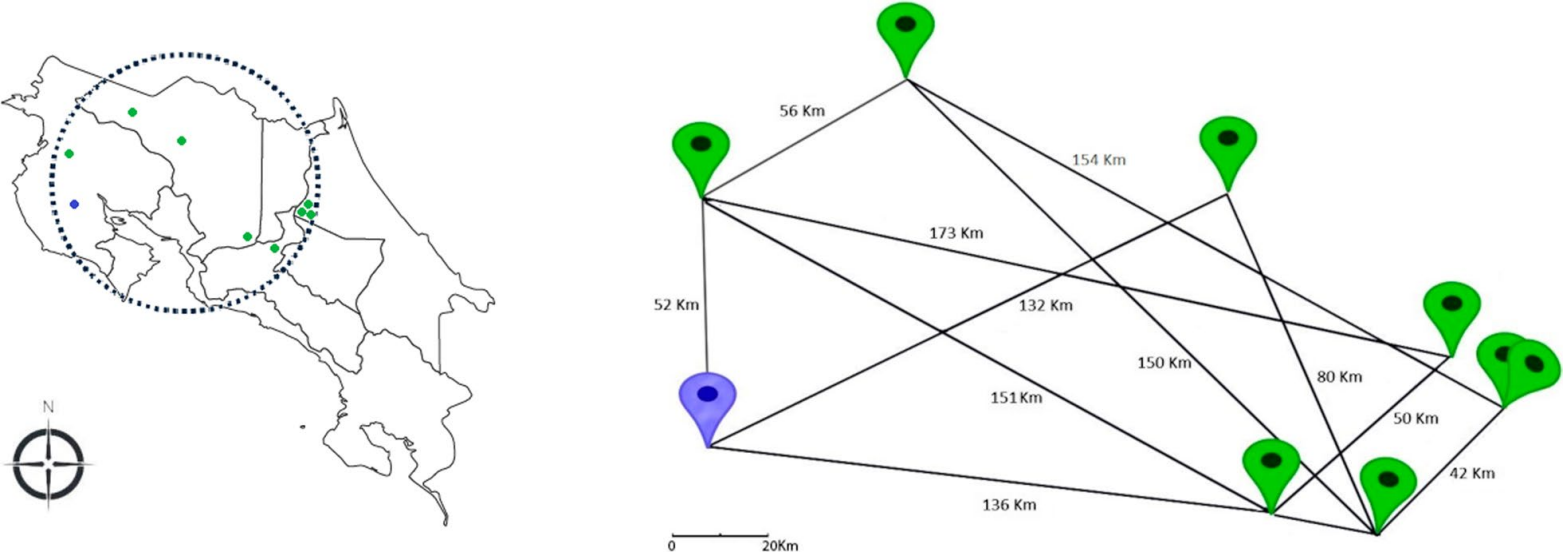
Geographic distribution of cases. Blue pin indicates the patient without the CLN6 Costa Rican mutation

Most of the patients were born in and currently live in areas distant from San José, Costa Rica´s capital city. There was a fraternal relationship identified between two patients who live in the province of Limón. Nevertheless, there was no evidence suggesting the presence of specific geographic areas with a higher frequency of cases (Fig. 1). Likewise, no consanguineous relationships were reported between the parents of study participants.

Gait disorders, vision loss and speech disturbances were the most frequent initial manifestations registered in medical records. Patient´s specific neurological signs and symptoms can be observed in Tables 2 and 3. At the time of clinical diagnosis, 77.7% (n = 7) of the cases presented abnormalities in the ophthalmological assessment with 87.5% (n = 7) of study subjects presenting with altered visual potentials when such data were collected. The majority of study participants showed abnormalities in the EEG and neuroimaging tests, in which cortical, subcortical, and cerebellar atrophies were common (Table 2).

**Table 2:** Clinical manifestations and evaluations at diagnosis

Patient codification	A	B	C	D	E	F	G	H	I
Main clinical manifestations	Behavioral and walking disturbances (ataxic)	Personality and behavior disturbances Seizures (generalized clonic) Extrapyramidal symptoms	Behavioral, walking, motor skills, language and sleep alterations	Alterations of motor skills and the ability to walk Cognitive decline Discrete dyspraxia	Alterations of motor skills and the ability to walk	Alterations of motor skills and the ability to walk	Alterations of walking ability (recurrent falls) Myoclonic seizures Cognitive decline	Visual and walking alterations Attentional difficulties Cognitive decline	Walking and language alterations Myoclonic seizures Attentional problems Loss of postural control
Opthalmological evaluation (OE)	Normal	Bull’s eye maculopathy, retinal vessels atrophy, retinal pigment epithelium and retinal alteration	Severe macular and retinal pigment epithelium alteration	Bull’s eye maculopathy	ON atrophy	Normal	Bilateral ONs atrophy ONs total pallor Generalized vascular thinning	Retinal atrophy with light discoloration	Bilateral ONs atrophy Retinal pigment epithelium and retinal alterations Generalized vascular thinning, macula with xanthophyll pigment
Visually evoked potentials (VEP)	Normal	Lack of response	Lack of response	Myelinopathic disorder	Diffuse axonal injury	Axonal disorder in associative areas	Diffuse axonal injury	Axonal disorder in associative areas	ND
ERG	ND	Impossible to perform	ND	Abolished	ND	ND	ND	Abolished	ND
EEG	Paroxysmal epileptic foci in left parietotemporal and right frontal lobes	Paroxysmal epileptic foci in anterior frontal lobes	Bursts of bilateral activity 4 Hz frequency during sleep and wakefulness Bursts of β activity in anterior regions	Disorganized patterns, focal slow right activity with high voltage bursts. Interictal epileptiform discharges	Absence of paroxysmal epileptic foci, slow baseline activity with generalized rapid rhythmic discharges	Absence of paroxysmal epileptic foci, slow posterior activity	Slow and diffuse baseline activity with greater amplitude in the posterior quadrant	ND	Interictal epileptiform discharges
Medical images	CT: cortical, subcortical and cerebellar atrophy	CT: cortical atrophy and diffuse subcortical atrophy	CT: cortical atrophy and diffuse subcortical atrophy	MRI: Generalized cerebral and cerebellar atrophy	MRI: severe cortical atrophy	MRI: diffuse cerebral atrophy and severe cerebellar atrophy	ND	MRI: Diffuse cerebral and cerebellar atrophy	MRI: diffuse cerebral atrophy and severe cerebellar atrophy

*ERG* electroretinogram, *EEG* electroencephalogram, *ND* no data, *ON* optic nerve, *CT* computerized tomography, *MRI* magnetic resonance imaging

**Table 3:** Most recent neurological examination findings and current treatment

Patient codification	A	B	C	D	E	F	G	H	I
Neurological examination: motor, consciousness level, visual alterations, epilepsy, functional level	Spastic tetraparesis, Somnolence, Complete lack of visual response, bedridden	Spastic tetraparesis, Somnolence, Lack of visual response to light stimuli, pupil deformity	Increased muscle tone in lower limbs, Somnolence, Blindness, Dyspraxia, Wheelchair bound	General hypotonia, Complete lack of visual response, Unstable in sitting position	Trunk ataxia, Axial hypotonia, Limb hypertonia, Lack of visual response	Tone and muscle strength preserved, Alert, Normal eye contact, Inability to walk	Spastic quadriparesis, Nistagmus, Focal Myoclonus, Beddriden	Lack of visual response, Right clonus, Able to stand in upright position using both upper limbs	Spastic quadriparesis, Myoclonic seizures
Other clinical findings				Dysphagia (nasogastric tube)	Dysphagia				
Treatment	Valproic acid Carbamazepine	Valproic acid Carbamazepine Topiramate	Valproic acid Clonazepam	Valproic acid Clonazepam Levetiracetam	Valproic acid Clonazepam Haloperidol Melatonin	Valproic acid Clobazam	Valproic acid Carbamazepine	Carbamazepine, Clonazepam L-carnitine Thiamine Biotin CoQ10	Carbamazepine Clonazepam Primidone Melatonin

The review of subject´s medical records allowed for a qualitative comparison between the initial clinical manifestations and the findings from the most recent neurological examination, showing a substantial deterioration in physical and functional capabilities over the observation period (Table 3). Valproic acid, carbamazepine and clonazepam were the medications mostly prescribed for the treatment of seizures.

In study patient A, genetic sequencing did not identify the Costa Rican mutation to confirm variant late infantile NCL. Full mutation analysis including: CLN1/PPT1, CLN2/TPP1, CLN3, CLN5, CLN6, CLN7, CLN8 and CLN10/CTSD was suggested for this patient. However, currently our Institution does not have the possibility to extend further genetic testing. After receiving the negative results for this patient new tests were performed and no data supporting alterations in metabolic or biochemical parameters was identified. Genetic confirmation of progressive leukoencephalopathy was also recommended. Currently the patient is receiving palliative care.

## Discussion

Worldwide, it has been reported that the initial clinical manifestations in CLN6 disease occur between 2 and 4 years of age [13], however in this investigation, the cases analyzed trended towards the older end of this range with regards to symptom onset (3.9 ± 0.6), with a range between 3 and 5 years of age. This may be due to the small number of cases studied, or possibly to certain variations in the clinical course of the disease linked to this causative mutation as has previously been reported for Costa Rican patients [20]. The age of diagnosis in this study was an average of seven years. This could be due in part to the challenges associated with diagnosis of a complex rare disease in a locality with limited utilization of genetic testing. In this patient cohort, females outnumbered males 2:1. This level of sex disparity has not been reported in clinical literature and is likely due to the small sample size. Differences in severity between the sexes have been documented for some forms of NCL, with more aggressive forms commonly reported in female patients, however this was not recapitulated in our study [29, 30].

The genetic alterations identified in the study population, pointedly the Costa Rican mutation and the substitution polymorphism in intron 2, have been previously reported [4, 22]. This substitution polymorphism has also been described in Argentinian populations [4, 28]. In the Costa Rican Batten disease population, the homozygous mutation and the presence of the polymorphism suggests that the polymorphism and the damaging mutation are inherited as a haplotype. Thus, unlike many other NCL mutations, the Costa Rican c.214G> T mutation has been associated with the founder effect [20, 31]. It is important to mention, that despite not having carried out multi-generation genealogical studies, the presence of the specific haplotype in all study samples, the absence of consanguinity registered in medical records and the varied geographical locations where study subjects were born and live, supports this local founder effect for this allele. It has been suggested that this founder effect was introduced in the initial pool of genes from the time of the Spanish and Portuguese colonization. This hypothesis has been mentioned in other medical conditions described in our country such as Wilson´s Disease [32, 33].

The main signs and symptoms observed in study participants generally agree with what has been reported worldwide for CLN6 patients, where visual loss, the presence of rapid and involuntary muscle movements, seizures, ataxia, and mental and motor deterioration are the most frequent findings described [16, 17]. Specifically, the published literature establishes that motor regression constitutes one of the first clinical manifestations [15], an aspect confirmed in our study subjects, where gait disturbances and alterations of motor skills were near constant findings in the initial stages of the disease (Table 2).

Where neuroimaging studies were available, cortical and / or subcortical atrophy were common findings, as has been consistently reported in NCLs [15, 17–19]. Regarding imaging modalities, magnetic resonance imaging (MRI) had limited availability in Costa Rica over the study period; many patient records thus only had computed tomography (CT) scans. Likewise, ERG equipment was not operational for the duration of the study period and ERG data is available for only three patients in the study. In the majority of the analyzed cases, deterioration was reported in both the ophthalmological evaluation and in the visual evoked potentials (defective macular light reflection, optic disc pallor, attenuation of retinal vessels, pigmentary retinal changes, macular degeneration and optic atrophy); all of these findings are congruent with what has been described previously in medical literature [34]. These changes can occur even before the onset of vision loss, which was documented in the present investigation. In this study, similar to what has been reported worldwide, substantial disease progression was observed when comparing the initial clinical manifestations reported in medical records with the findings of the most recent neurological examination [35, 36].

Current therapeutic options in this disease include symptom management for: seizures, sleep problems, extrapyramidal symptoms, behavior problems, anxiety and psychosis. Seizures in NCLs are often refractory to treatment and require the prescription of multiple antiseizure medications such as sodium valproate, lamotrigine, topiramate, levetiracetam, carbamazepine, and benzodiazepine derivatives [37, 38]. There were no major differences regarding the main antiseizure medications prescribed when compared to other countries, and possible variations would be due mainly to the availability of the medication in the country and in the social security health care system (CCSS).

Regarding the study participant in whom the c.214G>T Costa Rican mutation was not identified, it is possible that mutations in other NCL genes could be responsible for  the symptoms, as has been documented in other Costa Rican patients [20, 22]. It is worth noting that this was the only patient that presented with a normal ophthalmic evaluation and normal visual evoked potential at diagnosis, demonstrative of some disparity in their disease presentation. Additionally, while vast majority of Costa Rican patients with NCL are treated in the HNN, the possibility (although low) of patients in other regional or private healthcare centers cannot be disregarded.

## Conclusion

Collectively, this investigation confirms the presence of the c.214G>T Costa Rican mutation in most Costa Rican patients with clinical symptoms of NCL and sustains that the clinical characteristics of these patients are similar to those described in other regions globally.

## Data Availability

The datasets generated and/or analyzed during the current study are not publicly available due to internal policies of the CCSS that prohibited the publication of any patient’s information in any website outside of its network, but are available from the corresponding author on reasonable request.

## Abbreviations

CLN6: Neuronal Ceroid Lipofuscinosis Type 6
CCSS: Costa Rica´s Social Security System
NCLs: Neuronal ceroid lipofuscinoses
HNN: Costa Rica’s National Children’s Hospital
DNA: Deoxyribonucleic acid
ER: Endoplasmic reticulum
NGS: Next generation sequencing
SD: South Dakota
USA: United States of America
EEG: Electroencephalogram
ERG: Electroretinogram
ND: NO data
ON: Optic nerve
CT: Computerized tomography
VEP: Visually evoked potentials
APRONEP: Costa Rica’s Association for Children with Progressive Neurological Diseases
